# The Formation of Self-Organized Domain Structures at Non-Polar Cuts of Lithium Niobate as a Result of Local Switching by an SPM Tip

**DOI:** 10.3390/ma10101143

**Published:** 2017-09-28

**Authors:** Anton Turygin, Denis Alikin, Yury Alikin, Vladimir Shur

**Affiliations:** School of Natural Sciences and Mathematics, Ural Federal University, Ekaterinburg 620000, Russia; anton.turygin@urfu.ru (A.T.); denis.alikin@urfu.ru (D.A.); yury.alikin@urfu.ru (Y.A.)

**Keywords:** domain structure, self-organization, piezoelectric force microscopy, non-polar cut

## Abstract

We have studied experimentally the interaction of isolated needle-like domains created in an array via local switching using a biased scanning probe microscope (SPM) tip and visualized via piezoelectric force microscopy (PFM) at the non-polar cuts of MgO-doped lithium niobate (MgOLN) crystals. It has been found that the domain interaction leads to the intermittent quasiperiodic and chaotic behavior of the domain length in the array in a manner similar to that of polar cuts, but with greater spacing between the points of bias application and voltage amplitudes. It has also been found that the polarization reversal at the non-polar cuts and domain interaction significantly depend on humidity. The spatial distribution of the surface potential measured by Kelvin probe force microscopy in the vicinity of the charged domain walls revealed the decrease of the domain length as a result of the partial backswitching after pulse termination. The phase diagram of switching behavior as a function of tip voltage and spacing between the points of bias application has been plotted. The obtained results provide new insight into the problem of the domain interaction during forward growth and can provide a basis for useful application in nanodomain engineering and development of non-linear optical frequency converters, data storage, and computing devices.

## 1. Introduction

Local polarization switching via the biased conductive tip of a scanning probe microscope (SPM) has been studied since the first experimental realization of piezoresponse force microscopy (PFM) [1,2]. Further, the method has been applied to various uniform and non-uniform ferroelectric materials, such as the polar cuts of lithium niobate (LN), lithium tantalate [3,4,5], and barium titanate [6] crystals, lead zirconate titanate (PZT) thin films [7,8], and bulk ferroelectric ceramics [9]. The method has been demonstrated as a powerful tool for analyzing the domain growth in the presence of defects and in the non-uniform ferroelectrics [10]. On the other hand, polarization switching in a model uniaxial LN single crystal with a comparatively simple domain structure opens an opportunity for investigation of the ferroelectric domain evolution at the nanoscale. Several important effects have been studied recently, such as (1) domain size dependence on the duration and amplitude of the switching pulses [3]; (2) backswitching and anomalous switching [11]; and (3) the influence of the residual depolarization field on the domain kinetics [12]. A topic of special interest is the electrostatic interaction of isolated domains situated in arrays and matrixes, because this effect determines the shape of growing domains.

A recent study of the local switching at the polar cut of lithium niobate crystals allowed to reveal various regimes of domain interaction depending on the switching parameters: temperature, humidity, applied bias, and the spacing between the points of bias application [13]. It was demonstrated that polarization switching in ferroelectric materials under the action of a biased SPM tip can give a rise to complex spatiotemporal dynamics, including intermittency, quasiperiodicity, and chaos [13]. The attractive prospect of using this effect for systems of modern data storage and analysis was discussed [13]. This effect has been attributed to residual depolarization fields that appear as a result of screening retardation, taking into account the existence of the intrinsic or artificial surface dielectric gap (“dead layer”) [14,15].

It must be stressed that local switching by a biased SPM tip at thick polar cut LN plates creates needle-like domains of a sub-micron diameter with the charged domain walls [12]. The known experimental methods did not allow the visualization of these domains in the bulk with the required spatial resolution. This problem can be solved only by using local switching on nonpolar cuts of LN [16,17]. We recently showed that the needle-like domains formed in the surface layer of LN crystals under the action of the biased SPM tip can be visualized by PFM with high spatial resolution [16,17]. 

The studied domain interaction has also been described in the correlated nucleation effect, which led to discrete domain switching [18,19] and the formation of quasiperiodic nanodomain patterns and dendrite domain structures in various uniaxial ferroelectrics [20]. The obtained knowledge can be applied for the development of micro- and nanodomain engineering, which is widely used for improvement of dielectric, piezoelectric, and non-linear optical characteristics of the ferroelectric materials [21]. 

Here, we have experimentally studied the length change of interacting needle-like domains created by a biased SPM tip and visualized by PFM at the non-polar cut of MgO-doped lithium niobate (MgOLN) crystals. It has been revealed that the domain interaction leads to effects that are similar to those of polar cuts, but with the greater spacing between the points of bias application and voltage amplitudes. The decrease in domain length as a result of partial backswitching after pulse termination has been measured by Kelvin probe force microscopy (KPFM). We separated three main regimes of domain interaction—***uniform***, ***intermittent quasiperiodic***, and ***chaotic***—and plotted the phase diagram of the switching behavior as a function of tip voltage and the spacing between the points of bias application. The obtained results provide new insight into the problem of domain interaction during forward growth and can provide a basis for the useful application of nanodomain engineering and development of non-linear optical frequency converters, data storage, and computing devices.

## 2. Materials and Methods

The choice of the studied material (congruent lithium niobate doped by 5 mol % MgO) is due to the low switching threshold fields and speedup of the bulk screening process as a result of doping. Moreover, the essential increase of the bulk conductivity leads to a larger optical damage threshold, thus stimulating the wide application of MgOLN in various nonlinear optical devices [21]. 

Investigated samples (15 mm × 10 mm) of MgOLN were cut from 1-mm-thick wafers with a surface roughness of about 1 nm (Yamaju Ceramics, Owariasahi, Japan). The bottom surface was glued to the conductive substrate by silver paste.

The arrays of isolated needle-like domains were created by the application of a series of rectangle voltage pulses using a conductive SPM tip (National Instruments, Austin, TX, USA) with a constant distance between switching points. We used a pulse duration of 500 ms and amplitudes ranging from 60 to 140 V. The pulses were generated using an NI-6251 multifunction Data Acquisition Board (National Instruments, Austin, TX, USA) and high-voltage amplifier Trek-677B (TREK, Inc., Lockport, NY, USA). 

The domain structure was visualized using Probe NanoLaboratory NTEGRA Aura (NT-MDT Spectrum Instruments, Zelenograd, Russia) in PFM mode. Commercial probes NSC18 with a titanium–platinum conductive coating (MikroMash, Tallin, Estonia) with a radius of curvature R = 35 nm, resonance frequency f = 70 kHz, and spring constant k = 3.5 N/m were used. The piezoresponse measurements were realized by the application of 1–2 V AC voltage with a frequency close to the contact resonance. The spatial distribution of the surface potential was measured by a closed loop two-pass KPFM with the 0.1 V AC voltage applied to the tip and a 100 nm tip–surface distance at the second pass. The variable relative humidity was controlled using a homemade air humidifier. 

## 3. Results and Discussion

**Figure 1:**
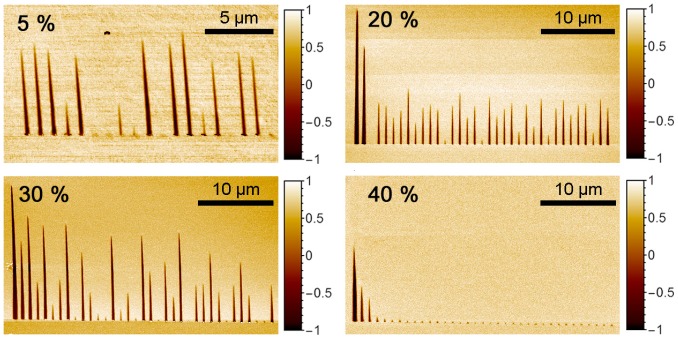
Piezoelectric force microscopy (PFM) images of the domain series produced by the local polarization reversal on the Y-cut of MgOLN at different relative humidities. Spacing between the points of bias application was 1 µm, and the pulse amplitude was 100 V.

In order to study domain–domain interactions at the non-polar cut, we wrote the arrays of isolated needle-like domains in line with various periods. Decrease in the spacing between the points of bias application allowed us to find that, for short enough periods, the length of the domains in the arrays essentially changed (the lengths of the neighboring domains were not equal to each other) (Figure 1). 

According to the results of studying the local polarization switching at the polar cut, we expected the domain sizes to decrease as the relative humidity increased, due to the formation of the water meniscus on the SPM tip [13]. We showed that a high relative humidity leads to the formation of a conductive surface layer [12]. The external screening of the depolarization field was stimulated, and the backswitching process was thus relieved [12]. 

We found that increasing the relative humidity up to 40% results in complete domain backswitching (disappearance of the switched domains) after pulse termination (Figure 1). 

**Figure 2:**
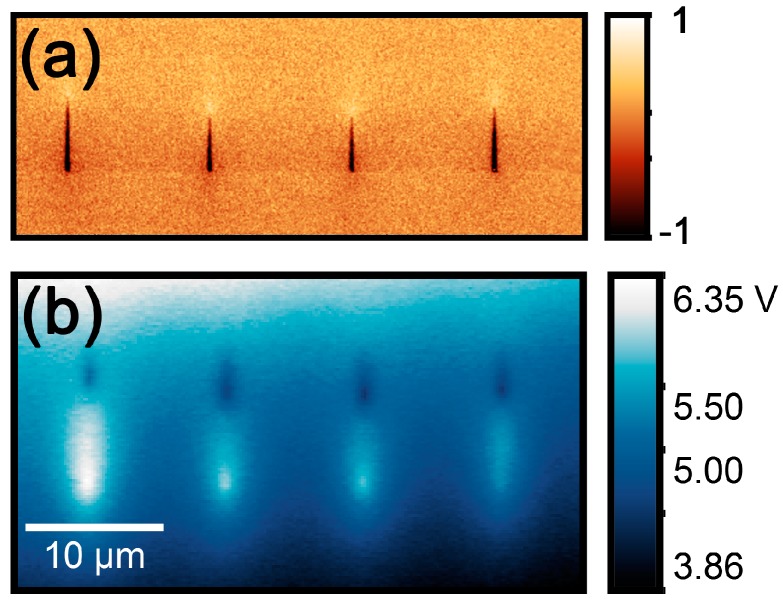
Domain arrays produced by the local polarization reversal on the Y-cut of MgOLN at the 5% relative humidity: (**a**) PFM image; (**b**) KPFM image. Spacing between the points of bias application was 10 µm, and the pulse amplitude was 100 V. The scale is equal for (a,b).

Analysis of the electric field distribution by KPFM demonstrated the existence of the electric field produced by charged domain walls of the needle-like domains. The lower value of the surface potential in the area close to the domain end (Figure 2b) has been attributed to the charge compensation during partial backswitching under the action of the residual depolarization field after pulse termination, which is the well-known final stage of the domain structure evolution during polarization switching [22].

We did not observe any significant differences between sizes of domains produced in dry air and with a relative humidity of about 20%. Moreover, we found that such humidity essentially decreased a general “background” of the surface potential caused by the spread of the injected charge. For this reason, all further experiments were conducted with a relative humidity of about 20%.

**Figure 3:**
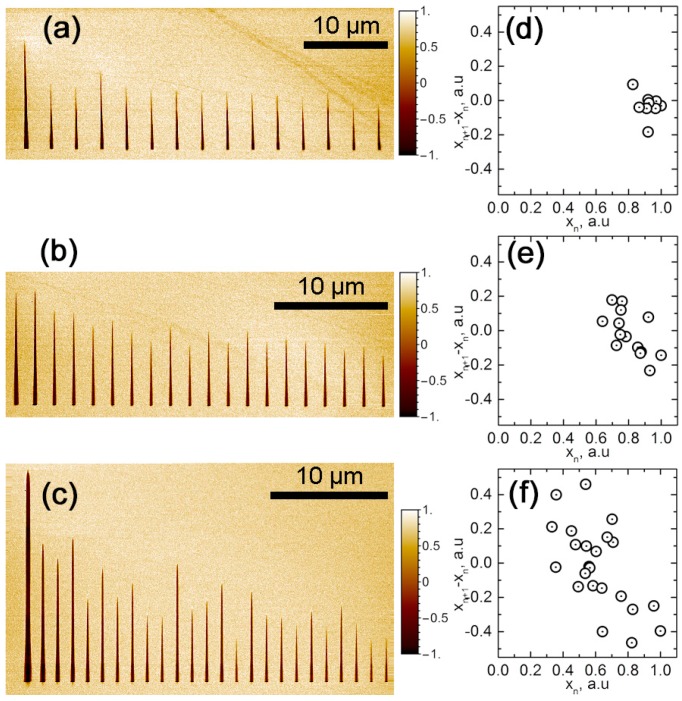
Domain arrays and plots of the difference in normalized lengths of neighboring n + 1 and n domains x_n+1_ − x_n_ versus the normalized length of n domain x_n_ for different spacing between the points of bias application: (**a**,**d**) 3 μm; (**b**,**e**) 1.7 μm; (**c**,**f**) 1.28 μm. Pulse amplitude: 100 V.

Analysis of the domain interaction revealed three regimes of the domain size distribution in the array, which were shown earlier during the polarization switching at the polar cuts of LN: (1) ***uniform*** (Figure 3a), with almost equal domain length; (2) ***intermittent quasiperiodic***, representing the period doubling (Figure 3b); and (3) ***chaotic*** (Figure 3c). The smooth decline in the domain length of the array is caused by the influence of the charge injected in the initial points of the bias application, which hampered further domain growth.

For the characterization of the different regimes, we plotted the normalized difference of the neighboring domains length versus the normalized length of one of them: x_n+1_ − x_n_ = q(x_n_), where x_n+1_ and x_n_ are the lengths of the previous and subsequent domains, respectively, and q is the empirical function, whose behavior depends on the domain interaction. 

For the ***uniform*** regime realized for the large spacing between the points of bias application and low voltage, the domain interaction function q(x_n_) is almost constant, which leads to the dense position of the experimental points at the plot (Figure 3d). For the ***intermittentquasiperiodic*** regime, the long pre-existent domains suppress the growth of new ones and the short domains relieve them; thus, the experimental dots at the plot are located over the elongated area (Figure 3e). A high applied bias or small spacing between the points of bias application lead to the ***chaotic*** regime and the wide distribution of the points at the plot (Figure 3f). 

**Figure 4:**
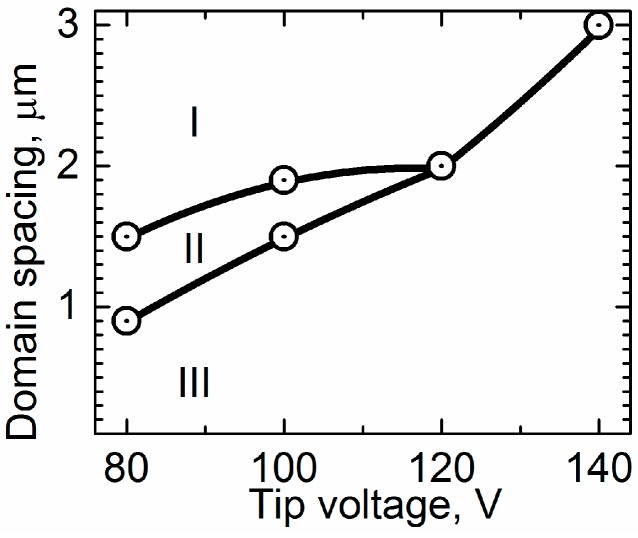
Phase diagram of the switching behavior (regimes of the domain size distribution in the array) as a function of bias (tip voltage) and the spacing between the points of bias application (domain spacing). Regions of various regimes are shown: (I) ***uniform***; (II) ***intermittent quasiperiodic***; (III) ***chaotic***.

The measurement of the switching behavior for the various pulse amplitudes (tip voltage) and spacing between the points of bias application (domain spacing) allowed for the phase diagram to be obtained (Figure 4). The phase diagrams at the polar and non-polar cuts are generally similar, but we found a significant difference in spacing and voltage amplitude, which can be attributed to a difference in the screening of the depolarization fields at the surface and in the bulk. It was found that, contrary to the switching on the polar surface [13], the ***merging*** regime was never obtained due to a suppression of the sideways domain wall motion. We also could not exclude the influence of the injected charge, which was able to supply a significant input to the domain interaction.

## 4. Conclusions

In conclusion, we experimentally studied the interaction of isolated domains at the non-polar cuts of the doped lithium niobate. It was found that the interaction was organized in a manner similar to that of polar cuts, but with different spacing and voltage amplitude characteristics. The polarization switching at the non-polar cuts of the lithium niobate and domain interaction were found to be significantly modified by the humidity, so we limited our studies to a relative humidity range of up to 20%. We separated three main regimes of interaction—***uniform***, ***intermittentquasiperiodic***, and ***chaotic***—and built a phase diagram. The obtained results provide new insight into the problem of domain interaction during forward growth and can provide a basis for the useful nanodomain engineering applications: optical and non-linear optical frequency converters, data storage, and computing devices.
